# Egocentric Chunking in the Predictive Brain: A Cognitive Basis of Expert Performance in High-Speed Sports

**DOI:** 10.3389/fnhum.2022.822887

**Published:** 2022-04-12

**Authors:** Otto Lappi

**Affiliations:** Cognitive Science/Traffic Research Unit (TRU)/TRUlab, University of Helsinki, Helsinki, Finland

**Keywords:** perceptual-cognitive expertise, sports, predictive processing, allocentric cognitive maps, wayfinding, race driving, egocentric perception, chunking

## Abstract

What principles and mechanisms allow humans to encode complex 3D information, and how can it be so fast, so accurately and so flexibly transformed into coordinated action? How do these processes work when developed to the limit of human physiological and cognitive capacity—as they are in high-speed sports, such as alpine skiing or motor racing? High-speed sports present not only physical challenges, but present some of the biggest perceptual-cognitive demands for the brain. The skill of these elite athletes is in many ways an attractive model for studying human performance “in the wild”, and its neurocognitive basis. This article presents a framework theory for how these abilities may be realized in high-speed sports. It draws on a careful analysis of the case of the motorsport athlete, as well as theoretical concepts from: (1) cognitive neuroscience of wayfinding, steering, and driving; (2) cognitive psychology of expertise; (3) cognitive modeling and machine learning; (4) human-in-the loop modellling in vehicle system dynamics and human performance engineering; (5) experimental research (in the laboratory and in the field) on human visual guidance. The distinctive contribution is the way these are integrated, and the concept of *chunking* is used in a novel way to analyze a high-speed sport. The mechanisms invoked are domain-general, and not specific to motorsport or the use of a particular type of vehicle (or any vehicle for that matter); the *egocentric chunking hypothesis* should therefore apply to any dynamic task that requires similar core skills. It offers a framework for neuroscientists, psychologists, engineers, and computer scientists working in the field of expert sports performance, and may be useful in translating fundamental research into theory-based insight and recommendations for improving real-world elite performance. Specific experimental predictions and applicability of the hypotheses to other sports are discussed.

## Introduction

Watching an elite athlete perform at the limits of human cognitive and physiological capacity is deeply impressive. To return a 200 kph tennis serve or drive a 200 kph race car the athlete must position themselves in space, and then summon up the correct motor programs precisely at the right time. The apparent ease with which a world-class expert can do this belies the fact that such performance is based on very sophisticated cognitive mechanisms honed by years of experience and practice.

High-speed sports present not only physical challenges but some of the biggest perceptual-cognitive challenges for the brain. The skill of these elite athletes is in many ways an attractive model for studying human performance in complex, dynamic real-world tasks, and their neurocognitive basis. One of the ultimate challenges for neuroscience is to understand the origins of such complex real-world skills.

What principles and mechanisms allow humans to encode complex 3D information, and how can it be so fast, so accurately and so flexibly transformed into coordinated action? How do these processes work when developed to the limit of human physiological and cognitive capacity? Such skills form a core perceptual-cognitive expertise in many sports, that more domain-specific techniques and processes then build up on. Understanding them needs a confluence of many fields of inquiry—and sometimes fragmented scientific literature.

**Figure 1:**
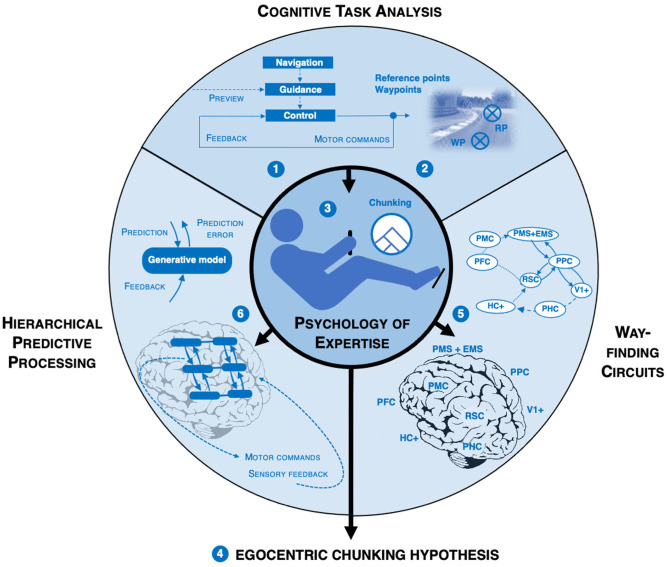
Overview of the concepts and hypotheses, and how they are logically organized. Also a road map to the way the article is structured. *Cognitive task analysis*, based on computational driver modeling in vehicle system dynamics engineering (including racecar engineering). **(1)** Specifically: the McRuer et al. (1977) *navigation-guidance-control* hierarchy that has also been used to understand higher-level cognitive involved in visual guidance in real-world driving (Lappi and Mole, 2018). **(2)** Interpretation of the hierarchical task analysis in light of domain expert knowledge elicitation, specifically the *reference point* concept (Lappi, 2018). *Waypoint* identification and interception characterizing the driving task (Lappi, 2014; Lappi and Mole, 2018). *Psychology of expertise*. **(3)** *Chunking* recurring patterns into long-term memory structures allow fast, automatic pattern recognition. Enables superior performance of experts despite strict capacity limitations of attention and working memory (Glaser, 1985; Gobet et al., 2001). **(4)** Chunking combined with waypoint identification and consideration of different frames of reference (Lappi, 2016) into the *egocentric chunking hypothesis*. **(5)** Interpretation of this hierarchical task structure/cognitive organization in terms of a neural hierarchy in cognitive neuroscience; *wayfinding circuits in the brain* involved in landmark-based navigation and representation of large-scale environments (Barry and Burgess, 2014; Epstein and Vass, 2014; Spiers and Barry, 2015; Epstein et al., 2017) and driving (Lappi, 2015; Navarro et al., 2021). **(6)** *Hierarchical predictive processing* (predictive brain) in cognitive modeling; the brain as an inference engine for stochastic estimation and control (Friston, 2009, 2010; Friston et al., 2012; Clark, 2013; on predictive processing in driving, see also Engström et al., 2018; Kujala and Lappi, 2021).

This article presents a proposal on how these abilities may be realized in high-speed sports, i.e., sports where the skill is based on controlling complex high-speed (self-) motion through a 3D environment. To this end, it brings together concepts and findings from a broad range of disciplines (see also Figure 1):

(1) Cognitive neuroscience of wayfinding, steering, and driving (neural circuits for wayfinding, visual guidance, and driving)
(2) Cognitive psychology of expertise (chunking)
(3) Cognitive modeling and machine learning (hierarchical predictive processing)
(4) Vehicle system dynamics and human performance engineering (hierarchical task analysis)
(5) Experimental research on human visual guidance, both in the laboratory and in the field (waypoint identification)

Its distinctive contribution is the novel way core concepts (*chunking, waypoints*, and *reference points*) get defined and used to analyze high-speed sports performance. The resulting *egocentric chunking hypothesis* is a specific proposal for how research in (1–5) can be extended to high-speed sports, and yield insight into human performance.

The hypothesis will be developed and illustrated within the context of the race driving. There are several reasons for this choice. First, the *task analysis* of a specific sport gives the hypotheses a level of detail and ecological validity (at least ecological face validity) that *grounds* the theory. Second, developing and illustrating the ideas in a single concrete domain ensures that the concepts will integrate and interlock in a way that stating them in more abstract and general terms would not. Third, using only a single domain (sport) to develop the ideas has the advantage that the propositions can then be corroborated or rejected, on the basis of observations from other domains. This way the generality of the statements can be genuinely stress-tested^1^. Finally, the case of the motorsport driver athlete is in many ways an ideal model for studying extreme human performance at the cognitive and physiological limits.

Arguments in favor of using race driving as the sport of choice are listed in Table 1. The reasoning here is that the preponderance of cognitive over physical determinants of performance in motorsport makes it a particularly good model domain to study for those interested in *cognitive* performance-differentiators in sports.

**Table 1:** Twelve features of race driving that make it an excellent environment to study elite performance.

I. *Performance differentiators point of view*:
1. Performance in high-speed vehicle-based locomotion depends on the information processing capacities of the brain, not the physical limits of the body. Differences in racing ability are not determined by the forces or power the athlete can generate, but skill in interpreting the situation, and dexterity in controlling the vehicle. 2. Unambiguous performance metrics can be used to objectively evaluate expert performance, with very high precision and ecological validity (e.g., lap time, various vehicle telemetry parameters). Here, well-developed domain-expert understanding can be leveraged to develop and quantitatively analyze and interpret performance metrics from driver performance engineering cf. e.g., Segers (2014).
II. *Measurement and modeling point of view*:
3. Representative tasks can be developed to span the full spectrum of simplified set-ups that can be used in the laboratory of brain imaging scanners through simulator set-ups of increasing fidelity, up to instrumenting real race cars for field data. This offers a unique chance to balance ecological validity and experimental control. 4. The spectrum from novice (everyday driver) to elite can be probed with the same task: taking a car through a bend is a task that even a novice can perform, yet it is meaningful and non-trivial for the expert (In many fields of expertise a task that meaningfully challenges an expert is impossible for a novice to perform). 5. Even expert skill in this domain can be distilled to very few degrees of freedom—all the knowledge and skill is basically expressed through the steering wheel, the brake, and the throttle pedal (and gaze control). 6. Compared to the biomechanics of the human body, the kinematics and dynamics of ground vehicles are very well understood (thanks to vehicle systems engineering). 7. The racetrack environment is a fixed 3D geometric layout and is generally quite stereotypical and sparse and therefore easy to model (compared to the fractal geometry and clutter in most natural environments).
III. *The theoretical analysis of its cognitive basis can leverage a large body of work on*:
8. Human driver modeling in psychology (Senders et al., 1967; Godthelp, 1986; Land, 1992; Land and Lee, 1994; Lappi and Mole, 2018). 9. Mature computational driver-in-the-loop simulation techniques in vehicle system dynamics engineering (McRuer et al., 1977; for review see Sharp et al., 2000; Macadam, 2003; Sharp and Peng, 2011; Keen and Cole, 2011, 2012; Johns and Cole, 2015; Nash et al., 2016). 10. Experimental psychology of the visual guidance of steering (Fajen and Warren, 2003; Salvucci and Gray, 2004; Wann and Wilkie, 2004; Warren, 2006; Wilkie et al., 2008; Fajen, 2013; for review see Regan and Gray, 2000; Wann and Land, 2000; Lappi, 2014). 11. The neural basis of steering (Field et al., 2007; Billington et al., 2010, 2013; Huang et al., 2015 and driving Bernardi et al., 2014; for review see Lappi, 2015; Navarro et al., 2018). 12. The neural basis of wayfinding in large-scale environments (Epstein and Vass, 2014; Barry and Burgess, 2014; Spiers and Barry, 2015; Epstein et al., 2017).

While racing drivers have to be physically conditioned to a very high degree (less so eSports athletes, as these are motorsports athletes who are not subjected to huge isometric loads or heat stress), consistent performance differences are not determined by physical conditioning (At the expert level you rarely lose a race, let alone get consistently out-qualified by 0.3 s—a 0.3% performance difference—because of being less fit). Because a racing driver uses the forces generated by a vehicle, performance is not limited by how hard, fast or long the athlete can produce forces on the environment. It is limited by differences in time-constrained judgment, timing, and precision regarding when and how to apply these forces. Here, sports, where performance is (to a large extent) limited by such information-processing capacities, are called *high-speed sports*, and the theory is meant to describe some determinants of human performance in those sports.

Also, the mature state of the art in measurement, modeling, and simulation techniques offers advantages for quantitative research, and the high level of development in AI techniques for eSports and autonomous racing makes human-machine comparisons timely, interesting and valid.

Yet, despite being a rich and potentially rigorous basis for understanding many aspects of performance, the skill of the racing driver has received surprisingly little consideration in the academic research literature (Potkanowicz and Mendel, 2013; Lappi, 2015; Ferguson, 2018). Certainly, this is the case if one compares the modest volume of research on racing to the thousands of articles on some other sports (such as football), or other forms of expertise with some similar features (such as music). Even compared to the research on everyday driving skills the study of expert driving performance is scant. It is hoped that this paper will inspire more work in this domain, and similar domains of human performance.

The article is organized as follows: Section “Navigation Guidance and Control: A Cognitive Hierarchy” lays out the core concepts, and how they are to be combined, according to the proposed hypothesis. Section “Neuroanatomical Basis in Wayfinding Circuits” outlines how the hypotheses align with the rapidly advancing research on the wayfinding circuitry of the brain, and Section “Egocentric Chunking as Predictive Processing” how it may be interpreted within a predictive processing framework. Theoretical implications and some open questions are taken up in Section “Discussion”, and novel experimental designs to test specific predictions and the range of applicability of the theory to other sports in Section “Conclusion”.

## Navigation Guidance and Control: A Cognitive Hierarchy

It is widely accepted that complex tasks generally (Lashley, 1951; Cooper and Shallice, 2000; Botvinick, 2008), and wayfinding in particular (Patai and Spiers, 2021), have a hierarchical (subgoal) organization. This hierarchical organization is often thought to be reflected in the hierarchical organization of the brain (Koechlin et al., 2003; Fuster, 2004; Botvinick, 2007).

**Figure 2:**
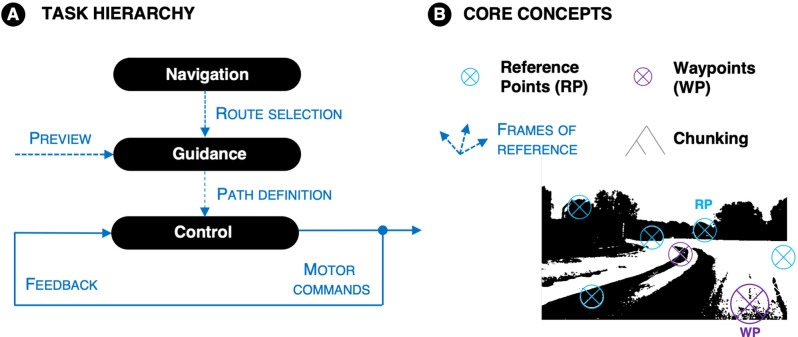
**(A)** The McRuer hierarchy. Navigation: choosing the desired path among alternatives. Guidance: definition of the desired future path based on visual preview. Control: steering and speed control by motor routines adapted to body/vehicle dynamics. Based on sensory feedback and feedforward control based on the top-down path definition. **(B)** Core concepts. Reference points: visual landmark features that can be recognized and used in self-localization. Waypoints: fixed objects or locations that are interception targets for self-motion. Frames of reference and transformations; different cognitive processes trade on information in different coordinate systems (allocentric, egocentric). Chunking: grouping elements into patterns that can be recognized and used to guide action. Expertise is based on having both more and bigger chunks stored in memory.

The hypotheses will be organized around a hierarchical analysis of the driving task, as is the standard approach in traffic psychology and engineering (cf. McRuer et al., 1977; Donges, 1978; Michon, 1985). In applied traffic psychology and ergonomics—the study of everyday driving in traffic—the standard approach is Michon’s classification of *Strategic, Tactical* and *Operational* levels (Michon, 1985; for a review analyzing relevant neuroscientific research in this framework see Navarro et al., 2018). In vehicle systems engineering—including racecar engineering—the hierarchy of *Navigation*, *Guidance*, and *Control* was proposed by McRuer and co-workers in the 1970s (McRuer et al., 1977; Figure 2A). The two classifications are similar but do not line up exactly^2^. Here the McRuer hierarchy is adopted (For a review analyzing relevant neuroscientific research in this framework see Lappi, 2015; for an analysis of race driving skills in this framework see Lappi, 2018; for its relation with neuroscientific research on visuomotor steering see Lappi and Mole, 2018).

• Navigation refers to *route selection* from among possible alternatives.
• Guidance refers to *path definition* based on *visual preview* information.
• Control is sensorimotor transformations from *feedback* to *motor commands* (feedback control), and the selection of* motor programs* based on the desired path defined at the guidance level (feedforward control).

The fundamental assumption the rest of the theory builds on is that the levels trade in different kinds of information—specifically in terms of:

• *Spatial scale*: Navigation dealing large-scale space including unobservable features of the environment, control with orientation to the immediate surroundings; guidance intermediate between these.
• *Temporal resolution*: Navigation dealing with stable, time-invariant landmark features and 3D space layout, control with the rapidly-shifting here-and-now; guidance intermediate between these.
• *Frames of reference: Allocentric* for navigation, *egocentric* for guidance, and *sensor/effector level* for control.

The terms “allocentric” and egocentric” are sometimes used in different ways. Here the distinction is taken to mean the following: an allocentric system stores information about the 3D environment in a form that is independent of observer motion or current point of vantage. That is: when the observer moves there is no need to update or modify any of the stored information (Apart from observer position. In other words: the allocentric spatial relationships between different locations in the world do not change. Only when the environment itself changes is there a need to *re-map*. Else only *localization* is needed).

### Cognitive Processes

Because they trade in different information and require different operations, the levels can be assigned specific cognitive processes, as studied in the experimental cognitive psychology of expert performance (These, in turn, should depend on increasingly well-understood neural circuitry, as studied in the cognitive neuroscience of way finding, Section “Neuroanatomical Basis in Wayfinding Circuits”, and be given expression in terms of hierarchical predictive processing, Section “Egocentric Chunking as Predictive Processing”).

The *cognitive processes* responsible for navigation, guidance, and control, in the present proposal, are listed in Table 2. The key novelty of the present work will be in: (i) interpretation of these processes (in the context of race driving) in terms of the core concepts of *reference points* and *waypoints*, and (ii) specifying how these processes (levels) are integrated vertically, by the core concept of *chunking* (Figure 2B).

**Table 2:** *Left column*: cognitive processes stated in the general, as described in the cognitive psychology and neuroscience of wayfinding and steering; *Right column*: specific operations according to the egocentric chunking hypothesis.

NAVIGATION	
**Cognitive Maps**	
• Establishing *long-term memory* for the stable (*time invariant*) and viewpoint independent *(allocentric)* structure of the environment. *Self-localization*	• Encoding *scene layout* and *landmark feature* information for *identifying reference points* and storing this information in long-term memory (“RP templates”). • Choice of “the racing line” (*desired future path* in relation to track knowledge based on reference points).
**Motor planning**
• Hierarchical goal structures, subgoaling, maintaining task set.	
*Reference points* for *chunking* (organizing egocentric representation of) space	
GUIDANCE	
**Maintaining an egocentric spatial image**
• Scene analysis based on *visual preview*. • Maintaining a dynamic (*time dependent*) and viewpoint dependent (*egocentric*) representation of the surrounding space (*spatial image* not limited to the *visual field*). Focusing attention on the spatial image. • Distributing covert visual attention (to a *peripheral visual field*) and overt attention (*gaze*) across time and space.	• Integrating localization information from memory with *visual preview* to *chunk* the visual scene into meaningful ensembles of scene elements (RPs and WPs): ° Dynamic tracking of *reference points* in the visual periphery (‘widescreen’ attention), gaze anchoring visual strategies. ˚ Determining directions and distances of *waypoints* to intercept (sometimes, but not always, by *gaze* fixation).
*Waypoint* identification for *chunking* (sequencing) actions	
CONTROL	
**Multisensory integration**
• Processing *sensory feedback* to estimate linear and rotational motions and accelerations (e.g., speed, g-forces).	• Timing actions for *waypoint* interception. • *Oculomotor* control (e.g., fixating waypoints to recover egocentric direction and distance and identify them as locomotor targets). • “Feel” for changes in frictional and inertial forces and vertical loads. “Balance”
**Motor programs**
• Action *timing*, sequential control. • Synergistic dependencies between effectors and sensors in motor coordination, active sampling of sensory feedback.	• Combination of *contact forces (on the pedals and the wheel)* to achieve *control forces* for “smooth” anticipatory steering and speed control. Motor coordination adapted to highly nonlinear body/vehicle dynamics.

Navigational route selection from among alternatives implies a representation wherein the different alternatives can be specified. These kinds of stable, enduring allocentric representations of large-scale environments are called *cognitive maps*. They provide a stable context for visual guidance, storing 3D layout information. It will be also assumed that cognitive maps store enough knowledge of the visual appearance of *landmarks* to allow recognizing them for landmark-based navigation^3^. For the racing driver, route alternatives could be the choice of different racing lines one could take through a bend^4^. This choice also depends on current goals and overall action plan (e.g., whether one is trying to take it easy on the tires, or do a qualifying lap). High-level *motor planning* (that is not involved with the specifics of motor pattern coordination) also belongs to the navigation level.

Guidance requires landmark *object recognition* and *visual scene analysis* to identify obstacles vs. clear space. The guidance level also involves *visuomotor coordination* involved in active gaze strategies, and the focus of attention across the (peripheral) visual field (i.e., covert attention). The guidance level is based on an *egocentric representation of perceptual space*; this is used for identifying, grouping, and tracking multiple elements in the *visual field* (Cavanagh and Alvarez, 2005) and remaining oriented in terms of a broader *spatial image* that extends beyond the current field of view (cf. Senders et al., 1967; Tatler and Land, 2011; Loomis et al., 2013). These processes deal with the use of *preview* information that is crucial in high-speed control.

Control has to do with the timing of actions, and the sensorimotor coordination of multiple effectors needed for coherent, synergistic action (referred to here collectively as *motor programs*). Bottom-up it is based on *multisensory feedback* and top-down on (visual) guidance, integrated by *internal models* of body dynamics (Wolpert and Kawato, 1998; Wolpert and Ghahramani, 2000; Wolpert et al., 2011).

### Reference Points

What information is represented in the allocentric maps in long-term memory? What parts of it are retrieved from memory, and how is it integrated into the egocentric visual field or spatial image?

**Figure 3:**
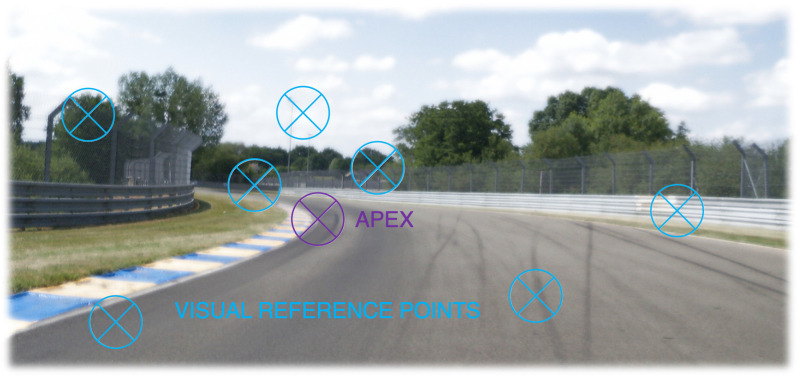
A racing driver will drive a familiar track on the basis of visual references. Fixing locations in memory and recognizing them by landmark features allows expert drivers to remain oriented, and drive at high speed even when available preview distance is limited. Where the references are will depend on the prior experience and individual preference of the driver [Note that cognitive modeling in other domains have estimated that the number of items in memory that would be needed to support expert performance would be on the order of tens or hundreds of thousands of stored relational patterns (chunks). As discussed in the main text, 10–100 *reference points* for 10–20 corners on a hundred racetracks would give the same order of magnitude].

The analysis that follows builds on the interpretation of perceptual-cognitive skills in race driving in Lappi (2018), which was based on analyzing domain expert knowledge, elicited from content analysis of technical training literature and fitted into the McRuer hierarchy (cf. also the related analysis of expert knowledge through verbal protocol elicitation in London taxi drivers in Griesbauer et al., 2021). It was found that one standard way for racing drivers to describe the track is in terms of *reference points* (Figure 3; see e.g., Code, 1983, 1986). Any fixed-in-place object or pattern as long as it can be clearly seen while driving at speed could be used as a landmark feature for a reference point. This could be a curb, differences in track surface texture, a gap in the painted edge line, a blotch of sand at the side of the track, a tree or a marshal post, or a different color safety barrier where the fence has been repaired, etc.

Identifying fixed reference points in the scene allows the racing driver to orient themselves on a familiar racetrack, and gives them the confidence they need. To “push” a race or rally car of a motorcycle to the limits, really intimate knowledge of the circuit committed to long-term memory is necessary. It is not possible to drive a racetrack at full speed based purely on the visual appearances of the bends—i.e., on the basis of available optical information. Many corners are blind (their exit cannot be seen while approaching them), and many small geometrical details such as the inclinations and camber of the asphalt and changes in grip levels cannot be visually resolved at speed in enough precision (A circuit racer will practice a track many times. A rally driver prepares pace notes, that are read by the navigator as an aid to memory. Neither takes the course “prima vista,” as it were).

The key observations here are that:

1. reference points have a stable location fixed in the 3D scene
2. they are “known” locations learned by reconnaissance (implying long-term memory), and
3. they can be reliably recognized based on visual features (they function as landmarks).

In terms of cognitive processes, this suggests that reference-point knowledge is stored in the driver’s cognitive map; this means both the stable spatial relationships between different reference points and their recognizable visual features. This is allocentric localization information at the navigation level and provides top-down route selection information.

On the other hand, the observed *egocentric* directions and distances to reference points also yield a *positional fix*. The driver remains oriented relative to the reference points. This means that in order to yield visual guidance (*path definition* for control), reference point knowledge must in some way connect to the array of visual features in *visual preview* (Egocentric chunking is a proposal as to *how* this connection is established).

### Waypoints

Reference point information gets stored in cognitive maps. *Waypoint* information is what connects it to procedural knowledge (motor processes and visual guidance). Information in cognitive maps needs to be retrieved to be of use in the online flow of action. The proposal is that actions are *sequenced* and *timed* for *intercepting waypoints*.

**Figure 4:**
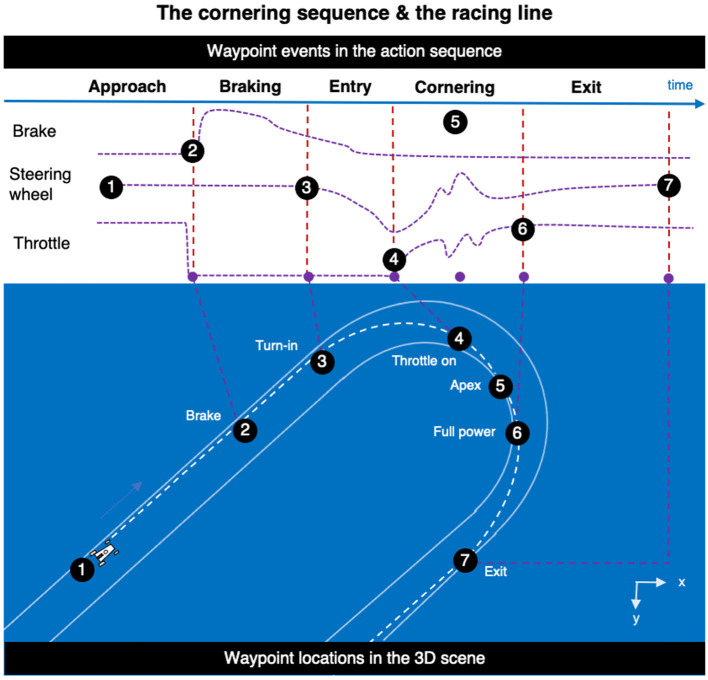
Waypoints. The basic cornering sequence can be partitioned into waypoint locations to sequence the racing line. These have a fixed location in the 3D scene. They are the locations at which critical control actions are performed. Operationally, waypoints can, therefore, be identified in vehicle telemetry time series on the basis of a small number of signals. What determines the placement of waypoints (for a given driver for a given bend), the mechanisms that *generate* the pattern of waypoint locations is the main concern of this article.

There are certain basic actions a racing driver performs in a bend (Figure 4), such as *braking, turning in, opening the throttle*, clipping the* apex* (cutting to the inside of the bend), getting on *full power*, and then *exiting*. Where these actions physically happen can be used to operationally determine *waypoint locations* on the track (From an operational point of view, waypoint locations are just locations where control actions happen. The theoretical question of how waypoint information is represented is precisely what the present theory is about).

Now, a waypoint, so defined, *need not be exactly at any visible reference point*. That is, a waypoint does not need to be a location that is designated by some specific visual feature or object at that specific spot. What is needed is that the reference points that surround the waypoint support the driver in deciding, fast and accurately, where to place the next waypoints.

But note that the known (remembered, mapped) locations of the reference points would not determine the location of the waypoint completely, top-down. The waypoint location *also* depends on the visual preview of the scene (presumably to make more precise estimates of egocentric distances and directions unfolding over time), as well as the current dynamic state (e.g., incoming feedback of tire grip and balance of the car). These sources of feedback can adjust the timing and amplitude of actions—*and thereby the waypoint locations—*in a bottom-up manner. Reference points have a fixed location in the scene, and reference point information was assumed to be stored in a *time-invariant* long-term memory representation—waypoints are more dynamic and adjusted on-the-fly.

#### What Is the Difference Between a Reference Point and a Waypoint?

The theory makes a sharp distinction between reference points and waypoints^5^. The use of *both* reference points and waypoints with different properties is a crucial posit of the theory and these two concepts should not be conflated. Roughly, reference points are something you see “out there.” They are fixed physical landmarks and recognizing them tells you “where things are” (and where you are). Waypoints tell you where and when to do things, relative to where you are now. They are not fixed by physical objects that are always “out there,” but by “where you want to go” right now.

While both reference points and waypoints are fixed locations out in the 3D scene (reference points more so, waypoints somewhat less so), their function and origins are quite different. They depend on different types of information at different temporal and spatial scales.

### Chunking

The final piece of the puzzle, the final core concept in the egocentric chunking hypothesis is the *chunk*. Theories based on chunking have been the predominant approach to expertise in cognitive science for the past 40 years (Miller, 1956; Simon, 1974; Newell and Rosenbloom, 1981; Gobet et al., 2001, 2016; Gilchrist, 2015). However, they have not been widely applied to sports performance or driving (applying them to race driving achieves both).

According to chunking theories, there are general psychological principles that apply in the organization of expert knowledge, regardless of the domain. Namely, when an expert in any field perceives, remembers, or creates a relation between elements of a situation (such as a configuration of chess pieces on a board being seen as a meaningful sub-pattern), a chunk is created in a *working memory*, and information about the pattern is stored into long-term memory.

With experience, fast and automatic *pattern recognition* based on this long-term memory will allow rapid encoding of complex situations with *a large number* of “small” scene elements into a *small* number of “large” chunks. Humans can only maintain attention on a few chunks at a time (3–4, and this capacity limitation seems to be a part of cognitive architecture, not substantially modifiable through training). But through increasing the size of chunks, a large amount of *information* can be rapidly encoded, even when working memory and attention have strict capacity limitations in terms of how many independent chunks that information can come in.

The basic hypothesis, then, rests on the following assumptions:

(1) There is a large body of spatial knowledge (reference points in allocentric cognitive maps)
(2) This supports fast pattern recognition of familiar scenes on a familiar racetrack (localization, positional fix based on preview).
(3) The number and size of chunks are a critical resource for expert performance in this domain (and a possible performance differentiator).
(4) Specifically, this happens by top-down control of motor programs by assigning egocentric waypoints to intercept.

**Figure 5:**
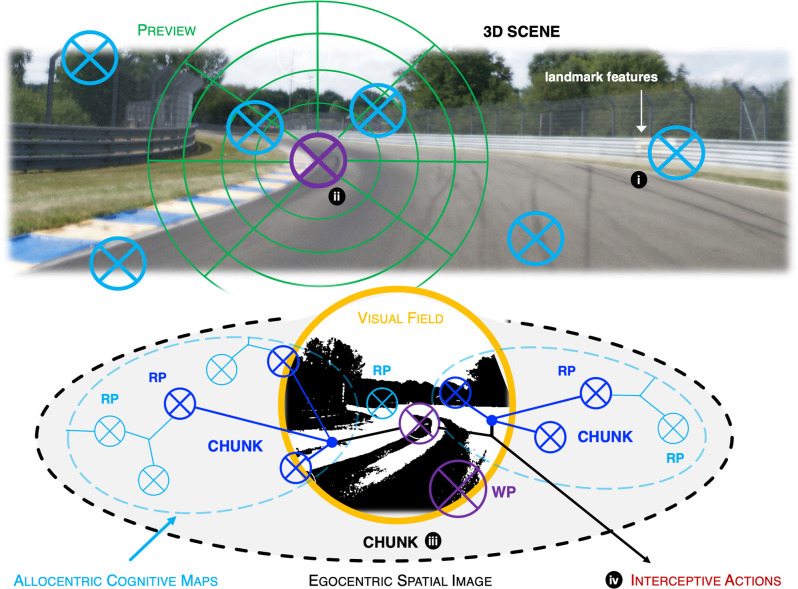
Schematic illustration of egocentric chunking of reference points and waypoints. **(i)** Reference points in the 3D scene are fixed objects or locations, recognized by landmark visual features. They function as landmarks for localization in the large-scale environment. RP information is stored into/recalled from spatial long-term memory that encodes their allocentric arrangement (cognitive maps). **(ii)** Waypoint locations on the path. They are identified in visual preview as targets of gaze or in peripheral vision. WP information is used to guide and sequence interceptive actions. **(iii)** RPs and WPs are chunked into relational patterns in the egocentric visual field/spatial image. **(iv)** Waypoints are targets of interceptive visual guidance, and timing cues for control actions at the waypoint. RPs allow the observer to localize themselves on the map; they are associated with WPs in the chunking process. Note that waypoints are not stored in allocentric memory, they are represented in *egocentric* space (relative to the observer). Waypoint locations are fixed locations in 3D space, but there need not be any reference point exactly at the waypoint; their location is based on the surrounding scene (reference points including but not limited to the current field of view), visual preview, and also current motor plans and goals. They are more flexible and dynamic than reference points, being associated with, and adjusted on the basis of, bottom-up feedback (For example, unlike reference points, the waypoint can be in different places on successive runs through the same bend as it is taken at a different speed, grip level, etc.). An action happening at the waypoint allows operationalization of the concept, even if there is no stimulus object or visual feature at that spot. Note that for illustration only a few RPs per chunk and only a few chunks are sketched out. In reality, the number of RPs per chunk is probably large—as is the number of chunks in long-term memory for any given bend. The number of chunks *active at any one time* is strictly limited by the attentional/working memory resource limits whereas the size of the chunks is not.

**Figure 6:**
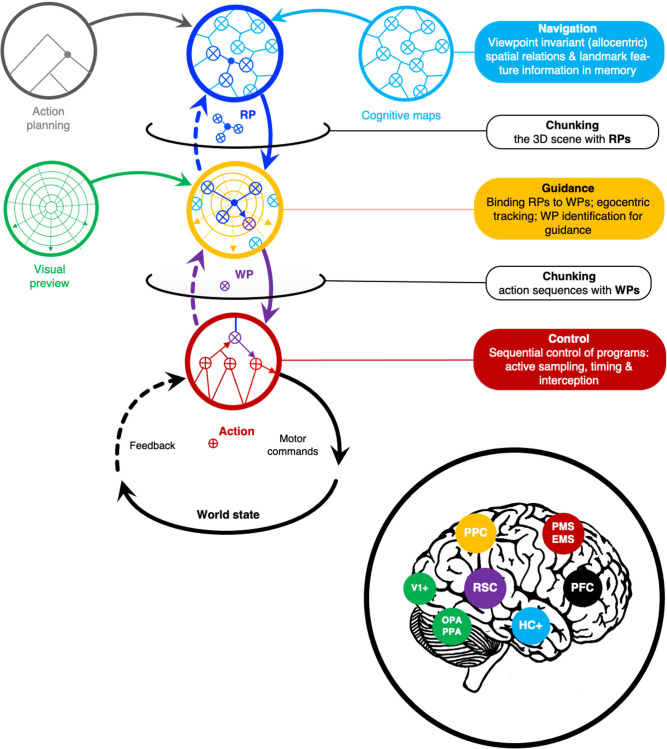
Main picture. Egocentric chunking as cognitive processes operating on representations at different levels of the hierarchy, in different coordinate systems, and at different spatial and temporal scales. Each level deals with different aspects of wayfinding and visual guidance (only). The core process is chunking WP and RP information at the guidance level. Waypoints are identified top-down by their association with visually identified scene elements (reference points in the cognitive map), but their precise egocentric positioning also depends on the preview of scene layout and bottom-up information (efference copy and integrated multisensory perceptual feedback from the control level). they are fixed “on the fly”. RP, reference point information. WP waypoint information. See also Table 2. *Inset*: Key nodes in the wayfinding networks (see Figure 7). HC+, hippocampal “cognitive map” system; PFC, prefrontal cortex; PPC, posterior parietal cortex; PMC, premotor cortex; PMS, EMS, pyramidal and extrapyramidal motor systems; OPA, PPA, occipital and parahippocampal place areas; MST, middle superior temporal, V5/MT; RSC, retrosplenial complex. The color-coding indicates the suggested mapping of the cognitive processes onto the network.

These yield the model outlined in Figures 5, 6; a conceptual framework that shows how the ideas and concepts will interlock in a specific way.

The egocentric chunking hypothesis states what sort of information the different levels of the hierarchy trade in and what kinds of processes operate on and pass that information between levels.

Reference points form a chunk of procedurally meaningful relational spatial information (depending on the current motor plan and bottom-up landmark recognition cues). This memory structure represents stable large-scale space well beyond the current field of view and localizes oneself in it (based on a positional fix from landmark distances and directions). It provides top-down information on the appearance of likely reference points and sends these predictions down to the guidance level. This handling reference-point information is *chunking space* in terms of allocentric and egocentric spatial relations aligned relative to one another.

From this information, predictions are made at the guidance level about the layout of the road ahead—including currently unseen parts of the circuit—e.g., the exit of a blind-entry corner (This top-down flow of information presumably also allows the driver to “visualize” the racing line, e.g., during mental imagery training). This top-down information is crucial in high-speed sports, because of the limited extent of visual preview.

The visual preview of the scene layout is the second input to the guidance level. The third input is bottom-up perceptual feedback and efference copy of the current motor program.

These are all combined into an egocentric representation (spatial image) that also contains waypoints to intercept. This path definition is top-down input to the control level for timing (cueing) the action at the waypoint. Each chunk of reference point information has to be associated with waypoints to guide locomotion. Waypoint information, however, emerges only as the result of chunking all three inputs: top-down reference points, visual preview, and bottom-up multisensory feedback (proprioceptive as well as visual), and efference copy.

## Neuroanatomical Basis in Wayfinding Circuits

**Figure 7:**
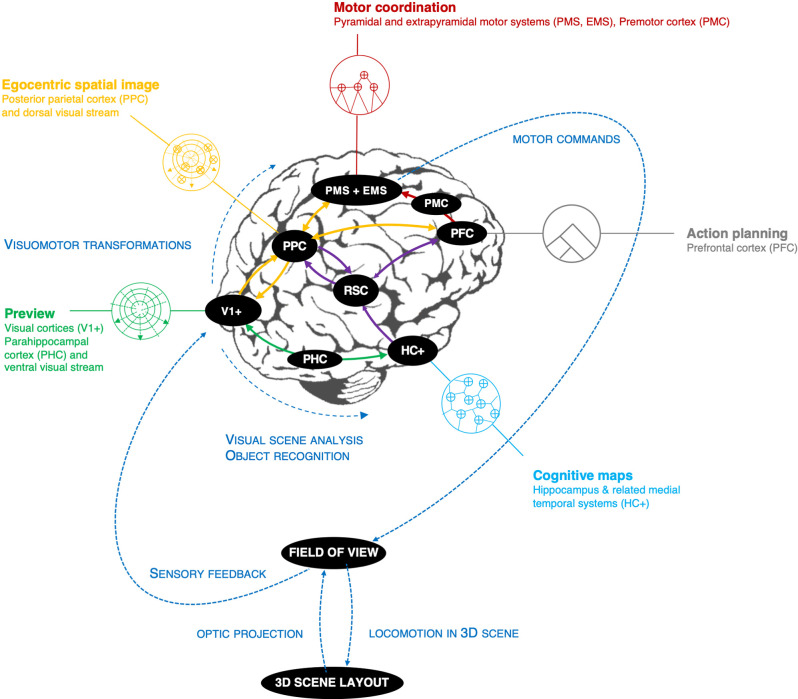
Wayfinding circuits, an approximate neural hierarchy that aligns with the information-processing hierarchy in Figure 6. PFC, prefrontal cortex; PMS + EMS, pyramidal and extrapyramidal motor systems (incl. related sensory systems); PPC, posterior parietal cortex; V1+ visual areas (here incl. parahippocampal and parietal place areas) S1+, somatosensory system; PHC, parahippocampal cortex; HC+, hippocampus and related medial temporal structures; RSC, retrosplenial complex.

Does the hierarchy of cognitive processes postulated above have a specific neural basis? This section discusses the neural organization of *wayfinding* circuits, and how the processes above may map to the brain. This is intended as a very rough overall mapping (Figure 7). The purpose is just to align the levels to the architecture with different levels in the neural hierarchy, from sensorimotor periphery to the higher-order brain areas, as needed to derive some predictions (in Section “Discussion”).

Reference points are stable spatial information stored in long-term memory. This sort of information is coarse, large-scale, and time invariant on a long time scale (an expert driver will be familiar with a track even years or decades after racing on it). This type of memory is dependent on the hippocampal system. Thus, *allocentric cognitive maps* at the navigation level would be supported by the hippocampus and related medial temporal lobe structures. These have been studied in landmark-based navigation tasks (Epstein et al., 2017).

*Motor planning* and “tactical” choice behavior is dependent on the prefrontal cortex (Patai and Spiers, 2021), which is also involved in the hierarchical subgoal organization of behavior and memory (Koechlin et al., 2003; Badre, 2008; Badre and D’Esposito, 2009).

Reference points and their arrangements in the scene are recognized through landmark visual features and geometric cues. *Object recognition* and visual analysis (Kravitz et al., 2013) of the *scene layout* (parahippocampal and occipital place areas; Epstein and Vass, 2014) are therefore essential for processing visual preview. Preview serves not only for bottom-up landmark (reference point) recognition but also maintains a sense of the scene layout, i.e., the egocentric but not purely visual representation of perceptual called “spatial image” (Loomis et al., 2013), “visual buffer” (Land and Furneaux, 1997) or “expectancy” (Näätänen and Summala, 1976). The key assumption of the present theory is that *this representation is organized into chunks, incorporating both reference points and waypoints*.

Somatotopic, retinotopic, and other sensorimotor information arrive in different coordinate systems and at different latencies. Integrating them requires complex coordinate transformations (Crawford et al., 2011). The posterior parietal cortex is thought to be involved in coordinate transformations for translating between different sensory and motor systems, to enable coordination of actions across different effectors (eye, hand, and locomotion) and maintain a coherent sense of space (Tatler and Land, 2011; Lappi, 2016).

Identifying, grouping, and tracking of elements in the visual field (Nummenmaa et al., 2017), tracking visual motion (V5/MT/MST: Born and Bradley, 2005), and visuomotor coordination (dorsal visual stream: Kravitz et al., 2011) including visual strategies for actively sampling the scene (gaze control networks, Lappi, 2016) and covert spatial attention (posterior parietal cortex and frontoparietal attention networks: Corbetta and Shulman, 2002; Corbetta et al., 2008; Ptak, 2012; Scolari et al., 2015) are also guidance level processes.

Control has to do with the timing of actions and the sensorimotor coordination of multiple effectors. This involves the pyramidal and extrapyramidal motor systems for manual and locomotor control, but also the lower, more reflex-like parts of the oculomotor system (fixation, saccade, blink control^6^). An important aspect of the control level is brain circuits relevant for (endogenous) timing of routine *motor sequences*, at different levels of complexity temporal scale (premotor cortex, basal ganglia, cerebellar circuits: Buhusi and Meck, 2005; Freestone and Church, 2016; Harrington and Jahanshahi, 2016; Raghavan et al., 2016).

### Chunking Circuits

The key to connecting the levels to one another is the concept of chunking; chunking allocentric/egocentric space with reference points (and motor plans) and chunking action sequences with waypoints.

The *retrosplenial complex* is thought to mediate between the stable, allocentric long-term memory information of the large-scale environment and the dynamic, egocentric information of the scene layout—“piecing together” snapshot views and “anchoring” the current scene to memory representations (Park and Chun, 2009; Vann et al., 2009; Epstein et al., 2017). This is therefore a key structure for the chunking space by reference points hypothesis. And indeed, it is larger in racing drivers, and moreover gray matter in RSC correlates with racing achievement (Bernardi et al., 2014).

The *ventrolateral prefrontal cortex* is implicated in chunking, in a number of task domains (Jeon, 2014). This structure is activated in racing drivers (but not control subjects) when viewing in-car footage of a racecar lapping a circuit (Bernardi et al., 2014).

## Egocentric Chunking as Predictive Processing

*Predictive processing* (a.k.a. predictive brain, or “Bayesian brain”) is a general theory of brain architecture that has resulted from a confluence of advances in machine learning and AI (which are producing concepts and methods that are increasingly useful for complex and naturalistic domains) and cognitive science (where these concepts are combined with experimental psychology and neuroscience methods, and used in computational cognitive modeling). It holds some promise for delivering unified theories of cognition (Friston, 2009, 2010, 2018; Friston et al., 2012; Clark, 2013).

It has been proposed as a useful way to analyze both driving behavior (Engström et al., 2018; Kujala and Lappi, 2021) and sports performance (Harris et al., 2021). Racing, of course, combines these.

### Hierarchical Predictive Processing

**Figure 8:**
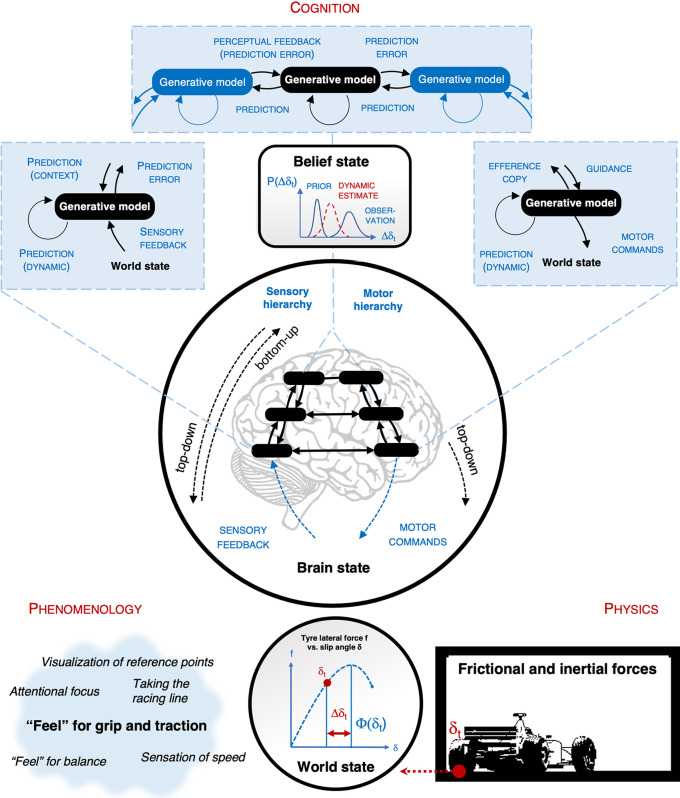
Hierarchical predictive processing. The brain as an inference engine for estimating the state of the world (the estimate is sometimes called “belief state”). For example: a racing driver’s brain may estimate how far a tire slip angle is from the slip angle that produces maximal lateral force. Physically this will depend on the vertical load and rotation of the tire—but it does not follow that the brain’s relevant generative model must represent these component factors, to veridically “simulate” the actual physics (And for each level it is sufficient if they can provide the adjacent layer “predictive” information that elicits no prediction error, there is no “master belief state” where it all comes together). Prediction is hierarchical, with a sensory hierarchy (posterior parts of the brain) and motor hierarchy (anterior parts). Prediction is based on generative models always with the same basic architecture: each of them **(i)** stores information about some aspects of the world (only) and their dynamics as priors, **(ii)** receives feedback. Please note that the term “feedback” is used in different ways in different literatures. Here *feedback* flows in what is anatomically called the *feedforward* direction, and also in control theory the guidance signals would be called *feedforward* control. In the predictive brain framework feedback refers to the “new” information entering the system (at any level), which drives internal model update. For example in a Bayesian framework the feedback is observational data (and the dynamic predictor is the likelihood, the context and the memory internal to the generative model are priors), **(iii)** and possibly receives context information (predictions). On the basis of the internal model and context information, new feedback is predicted and compared to actual feedback; prediction error is used to update the internal model. Prediction error (only) is passed forward, as feedback to the next layer (predictive coding). Note that in a Predictive Processing architecture there is no architectural distinction between perceptual, motor, and cognitive representations. Both the sensory hierarchy and motor hierarchy embody the same architecture all the way down. Perceptual feedback is prediction error; so are guidance and motor commands (prediction of motor program activation, and proprioception/muscle activation, respectively; on the motor side the prediction error drives the motor system, which is called active inference). Note also that on the sensory side prediction error is “bottom up” but on the motor side, it is “top-down”, i.e., in the direction towards the sensorimotor periphery.

According to the predictive processing view, the brain is a “predictive” inference engine (Figure 8). It generates perception, action and cognition by matching incoming *sensory input* with top-down expectations (*predictions*) of said input (Note that prediction here does not necessarily involve *forecasting* the future—it means predicting aspects of *feedback information* from the world that has not been observed yet, by a system at some given level of the hierarchy—the system may be “predicting” information about past events that it gains with some delay). The predictions are generated by a *hierarchy* of *generative internal models* that embody assumptions about the statistical regularities and causal relationships of the universe (including the body and the brain). The models are updated based on *prediction error* (difference of actual and predicted input). To save bandwith, onlyprediction error is passed up the hierarchy (*predictive coding*).

The internal models update in such a way as to better predict future inputs—learning at all levels is driven by minimization of prediction error. This *unconscious inference* process matches incoming feedback against prior information stored in the brain, maintaining and constantly updating internal *state-estimates* (sometimes called *belief state*) of relevant *state variables* (sometimes called the *world state*). Note that the term “belief” is used in a technical sense and should not be taken to connote explicit, conscious knowledge. Also, the world state is not the physical situation *per se*, but only whatever aspects of the physical situation the brain actually registers and keeps record of.^7^

Now, if these core processes involve developing and updating generative internal models, then such models and information embedded in them are available for model-based (predictive) control. This is particularly useful in high-speed tasks, like race driving, where feedback and motor delays are substantially large relative to the rate of events and the response frequency of the controlled system (McRuer et al., 1977; Macadam, 2003; Nash et al., 2016). A race car has a high-frequency, nonlinear response to the driver’s control inputs. At the highest speeds, responding to the observed car behavior would mean that the driver is constantly “behind” what the car is doing.

On the perceptual side, the lowest levels of the hierarchy predict sensory feedback, such as visual information from the retina (optic flow), haptic sensations from the skin (steering wheel self-aligning torque), proprioceptive organs in the joints and inner ear (g-forces, muscle tension, body and limb pose), ears (engine and tire noise), and the dynamic state of the vehicle (speed, oversteer/, understeer). On the motor side, the lowest level^8^ predicts motor routines (shifting gears, moving gaze) and the motor actions (arm and ankle movements, blinks, and saccades). The predicted motor actions themselves drive the neuromuscular system (This is called *active inference*, where the discrepancy between the predicted and the current motor action—prediction error—is minimized not by updating the motor representation but by the body actually performing the motor action to match the prediction).

### Chunking in the Predictive Brain

The core assumptions of the waypoint chunking hypothesis rely on a hierarchy of representations from the short-lived, local, and sensor/effector level (at the sensorimotor periphery) through an intermediate egocentric representation to stable, global, and allocentric cognitive maps (deep inside the brain, high in the neural hierarchy). Interpreting them in a predictive processing framework draws out a number of interesting implications, some of them counterintuitive.

The key is the fact that at all levels the generative models are attempting to predict their own inputs—*not* forecast discrete physical events in detail. *The lower-level information never gets all re-represented at the higher levels*. For example, situation-specific details of a waypoint *cannot* be accurately recovered from reference point information in memory. This has some consequences that are worth pointing out, as these are important characteristics of human expert performance:

(1) *An economy of representation (rather than a single master representation)*. There is no need for a Euclidean, “a high-definition internal simulation” of Newtonian motion in a cartesian coordinate system, for example. The higher levels do not need to be able to model (predict, control) the physical world as such—they only need to be able to model (predict, control) the level below them. Thus, *there is no single “high-definition simulation for the mind’s eye”*, where *all* the knowledge about the spatial world, self-motion, and action would come together for a homunculus to view in a “cartesian theatre” (Dennett, 1991). The higher levels need not represent the whole world/body dynamics, but only such aspects as are needed to issue (useful) predictions to the lower level. That is: the higher action planning and localization level representations can be left to deal with stable (time-invariant, viewpoint invariant) properties and frames of reference, while the lower levels can be left to deal with the details of motor execution and timing.
  For example, the navigation level needs to only be able to tell the guidance level what the reference points look like, and wherein the scene (roughly) they should be. Their exact egocentric location is left to the guidance level to figure out, on the basis of preview information that is *only* available at the guidance level but not at the navigation level. If there is a match, then the waypoint for the matching chunk is identified and positioned in an egocentric space. This information is passed down to the control level to achieve interception. The sensory consequences of interception can also be used as a timing cue for the next action in the sequence. But the sensory consequences are only predicted (from waypoint information and current feedback and motor efference) at the control level. They are not forecasts of events “out there” in navigational space. If top-down information guides the control level in ways that help achieve successful interception and no prediction error signal is elicited (and the sensory information is not relayed up to the guidance level).
(2) *Online adjustment of performance (rather than “metronomic” repetition).* This view of the way the navigation and guidance level work also has implications at the control level: skilled motor routines are not simply a metronomically rigid repetition of the exact muscle coordination patterns (control level). This may be significant for understanding the flexibility typical of human skill (Bernstein, 1967, 1996). The “feedforward” or top-down guidance of motor commands *does not flow from a motor program that would completely specify the necessary movements* as in the 1970s concept of a motor program (Keele, 1968; Schmidt, 1975, 2003; cf. precognitive control in McRuer et al., 1977), nor from an action plan that would completely specify 3D behavior (as if there were a cartesian, euclidean 3D replica of the world inside the brain, cf. Peer et al., 2020).
  The flexible placement of waypoints is an example of this. Information flows not only top-down from reference point memory but bottom-up too, so that the waypoint position (desired future path) at the guidance level gets *adjusted* on the basis of the current dynamic context. It is not determined by the cognitive map for the bend (and the high-level motor plan of how to take it).
(3) *The richness of spatial representation (rather than a bottom level of scene elements, given by rules)*. Because we are looking at a dynamic task in time and space, the economy of representation may not only be achieved through the dimension of abstraction of information but by making the information only available for use on a “just in time” basis. Consider this: the number of chunks in long-term memory is large (or more accurately template information from which chunks can be generated; Gobet et al., 2001). The capacity of attention and working memory limits processing to a few chunks at a time. But because the size of a chunk is unlimited, experts can transcend the limitations by taking in large amounts of information “at a glance”. Now, there are two ways to think about the size of a chunk. One can think of bigger, richer, chunks either in terms of “adding detail”—i.e., more reference points and waypoints in the current visual field (This would be like adding squares and/or pieces to the chessboard). Bur there is another, more interesting theoretical possibility: *recursive* chunking of chunks themselves.
  Referring back to Figure 5, this means that the elements in the sub-chunks (blue) will have the same internal structure as the chunks themselves, which has the same structure as the main chunk (purple). In the process of developing athletic expertise chunks for common environmental patterns can be “refined” to take ever subtler (spatial and temporal) detail. The basic elements and relations are not *immutable*. They do not “bottom out”. This is like making the details of the piece or a square relevant to how a chess piece can be used (information that is of course totally abstracted away in chess by making the explicit rules the ground level).

These characteristics may be important for understanding human performance generally, and also how human performance differs from machine performance (Section “Human vs. Machine Performance” and “The Human Advantage” below).

## Discussion

A proposal has been made on how the wide array of sometimes disparate approaches to (expert) driving performance may be brought together, under a unifying hypothesis: egocentric chunking based on reference points and waypoints. It is founded on a hierarchical view of the driving task (McRuer et al., 1977), and grounded in a careful task analysis based on driver training concepts and ideas, extracted through knowledge elicitation of the expert literature (origin of the concept of reference points; Lappi, 2018), and on the theory of waypoint identification based on experimental and modeling work in the visual strategies and steering models, both in psychology and engineering (Lappi and Mole, 2018). Through the concept of chunking, these are integrated with the psychology of expertise, and through the concept of different coordinate systems and coordinate transformations to visual neuroscience and the neuroscience of wayfinding. (Re)interpreting chunking as predictive processing is proposed as a fruitful way forward.

Next, some open questions, theoretical implications, and empirical predictions from the theory are discussed, as well as the question of generalizability to other sports.

### Is This an “Overly Cognitive” View of Sports?

The chunking concept gives a way to look at sports performance from an unusual perspective. It gives a more “cognitive” view of the skill than is perhaps typical in driving or sports research. Conversely, extending the chunking concept to sports is a way to look at chunking itself in a different light. But is this a too “cognitive” take of the expertise of the racing driver, borrowing the chunking concept from research in chess as it does, too heavy on “thinking” and too light on “raw skill”?

While there is of course a tendency to view chess, and other board games, as more “intellectual” tasks than driving and sports, which are seen more as “physical skill”, this sells short the cognitive challenge the brain must tackle in real-world expert performance (Walsh, 2014), and the complexity of information-processing and perceptual-cognitive skill in sports is easily underappreciated. On the other hand, the knowledge of experts is always highly *procedural* (Glaser, 1985), and the knowledge described here is *knowing what to do*, not necessarily kno*wing what to say*: *implicit* understanding of how to use the reference points to solve the problem posed by the 3D scene layout and vehicle dynamics—not how to think, reason or communicate *explicitly* about the relevant information^9^.

### Estimating the Number of Reference Points

How many reference points must get chunked to become an expert racing driver? It is a commonly held belief among domain experts that a skilled racing driver will have committed to memory a very large amount of information about reference points, and that an expert racing driver when learning a new track, will quickly pick up a very large number of useful reference points for each part of the track (e.g., Code, 1983, 1986; Bentley, 1998, 2003). Having lots of reference points allows the driver to choose their braking and turn-in points, and to place their car accurately and with confidence.

The exact number is not known, as there are no established methods of counting them (neither among domain experts nor in basic science). But a reasonable order-of-magnitude guess could be between ten and one hundred for each turn. The reasoning behind this estimate is as follows. Let us say there are half a dozen to 10 actions, or waypoint locations, for a bend and that each is associated with anything between 5 and 10 memorized reference points, which in some way aid in localization and timing. A typical modern circuit will have 10–20 bends. A professional driver may know hundreds of circuits. Therefore, if we think of the “cognitive map” for each turn as encoded into such an associative pattern of reference points, then this yields an estimate on the order of 10^4–^10^5^ such patterns. Or: on the order of tens of thousands of *chunks*. This is in fact similar in magnitude to classical estimates in chess expertise.^10^

### Chunking in Chess vs. Chunking in Sports

Early computational cognitive modeling of chess expertise (Chase and Simon, 1973a, b; Gobet and Simon, 1998) led to estimates on the number of chunks required to reach expert levels of memory and performance. These are on the order as much as tens of thousands of such stored relational patterns. The idea is that the more sub-patterns (recognizable chunks) the player has in long-term memory, the more likely it is that *some* novel configurations on the board will match many stored patterns. Working memory and attention have a capacity limitation of only a few chunks at a time, but there is no limit to the size of chunks—e.g., how many pieces can belong to a chunk. A large number of big chunks in long-term memory allows rapid encoding of a large number of scene elements (pieces) into a small number of big chunks. This allows the expert to circumvent the architectural limits of working memory by perceiving large “wholes” where the novice perceives separate “pieces”.

The term “chunk” originates from delayed recall experiments, where chess experts would grab a chunk of chess pieces, place them on the board in the appropriate squares, then pause to look at the board before grabbing another chunk of pieces, placing them, pausing, then grabbing another chunk of pieces. The basis of the chunking theory was the insight that the pieces were not being selected randomly. The pieces belonging to the same *chunk* form a single *meaningful*, interrelated sub-pattern on the chessboard (Say, a castled white king surrounded by a rook and a few pawns in a defensive array. Note that “defensive” here is a higher-level concept that relates to “lines of play” that are not directly specified by the rules of chess, or in terms of any single piece or square coordinate).

This brings us to this more subtle question of what is being chunked: in the real world, what are the “elements” to be chunked, in the first place? What are the natural elements in a *real-world visual scene*? What are the meaningful relations? How should one define a “chunk” of a racetrack? Of an alpine piste? Of a football field? The 3D scene of a racetrack does not come neatly separated into “reference points” and “racing lines”, the way chess positions do. The elements are not “given” (and they may never “bottom out”, see below). Reference points are simply *whatever objects or locations the scene gets chunked into*, and remembered as meaningful relational patterns.

By emphasizing chunks based around stable allocentric information is not implied that there should be a metrically accurate *replica* of the spatial world inside the brain. A cognitive map or a spatial image, into which physical waypoint locations and reference points are mapped, is a metaphor. Indeed, in understanding the “representation of the environment”, the more fundamental question is the converse: *what* physical locations are reference points or waypoint locations? Mapping the internal representation *back* onto the 3D scene.

In chess or other board games, the pieces constitute the basic elements to be chunked: *what* a piece is, and *how* it can be operated on is “given” and *immutable*, within the rules of the game. But in physical environments (sports) this is not the case: the 3D scene does not come automatically parsed into what “objects” or “locations” are relevant for actions. The athlete has to figure this out, from experience. And likewise, scientific theories to understand the expert athlete’s cognitive processes must figure is out as well. Indeed, if we could understand *which* elements are selected for “chunking” in a real-world scene, and *what* kinds of relations among them are stored, we could be one step closer to understanding why human athletes so comprehensively outperform robots (e.g., human racing drivers vs. autonomous racing cars).

### Human vs. Machine Performance and “the Human Advantage”

The human ability to master different physical tasks and conditions is beyond any existing, or immediately foreseeable, computational techniques. Although algorithms can be developed to perform at a “superhuman” level in computer-simulated environments (including specific motorsport eSports sub-tasks) and made to work in predictable real-world environments (including a race track), these systems are today still very much engineered for the specific application (And supported by teams of humans). In terms of the flexible use of physical means afforded by the environment and the body, humans outperform AI and robotics; expert athletes vastly so.

This “human advantage” over machines in dynamic real-world situations suggests that the brain may have ways for organizing perception, action, and memory that are different from current AI. This is of interest to the cognitive and neural sciences. One example is how expert human racing drivers can drive faster and more reliably than autonomous racing cars, and adapt to different tracks, vehicles, and conditions in ways that algorithms cannot.

This advantage stands in contrast to the way machines by far outperform humans in tasks like chess and go (provided there is a human being on-hand to physically move the pieces for them, that is…), and even computer games with complex dynamics. It is all the more remarkable in high-speed sports, considering sensory and motor delays imposed by strict physiological limitations on the human nervous system (on the order of hundreds of milliseconds).

While board games have been a very useful test environment to develop theories of expert performance, sports is arguably the more generalizable and interesting field—at least in terms of understanding the “human advantage”. Why? Consider the fact that the best chess algorithms have far surpassed the ability of the best human players…as long as “the game situation” remains restricted within the domain *defined by the rules of chess*. In a game such as chess, the rules directly and completely define the basic elements: the board structure, the allowed placement of pieces into coordinates, and the moves that are feasible explicitly specified and can be enumerated from a limited set of rules. What is being chunked is “given”. But in racing *or any other sport or real-world skill*, this is not the case.

*All the “real rules” in sports are unwritten*. For example, the written technical and sporting rules of a championship obviously are not about what the driver is allowed to use as reference points. Which stable objects or locations could or should be used as reference points is up to the racer’s brain to decide, based on some internal rules of what would make a good reference point.

Also, consider this: in a physical task there is in principle no level where the scene elements “bottom out”. In chess, once a piece is established as a pawn, the physical characteristics or its sub-parts need not be considered: they do not make a difference for “how it can be moved”. And once its coordinate is established in terms of the square it occupies, its exact positioning does not matter for the “lines of play” that can be generated. For physical performance, there is no such base level where the scene and task dynamics would “bottom out”. Potentially, minute differences 3D scene layout or in body dynamic state could make a difference to technique—how the body or the vehicle can be moved, what lines one can take through the environment. The elite athlete could be making use of these. Not only are the rules unwritten—*even the “real game board” is undefined!*

*It is the size (or depth) of chunks, not the number of chunks one can recognize at any one time, that separates the expert*, according to classical chunking theory. The elite athlete could be committing to memory more and more objects, locations, and movement patterns they afford, in ever finer and finer detail. The detail that can make a difference in the performance. What this means is that the size of a chunk *in terms of interrelated items* can grow without bound, in the course of becoming an elite athlete, as deeper, more detailed understanding of the spatial world, including the body, and more subtle fine-tuning of action is developed.

### Empirical Predictions

This section outlines experimental paradigms to test the assumptions. Note that while the concepts were developed and illustrated within the example of race driving, most of the research questions and paradigms sketched could be developed for similar sports, or using experts from other domains, provided the task demands are similar.

#### Expert Knowledge Elicitation

Some of the ideas are based on a task analysis based on domain expert knowledge elicitation from driver training literature (Lappi, 2018). This is particularly so when it comes to reference points. More direct experimental work on visual strategies and especially direct tests for expert track memory are needed to extend and validate some of the assumptions.

Similar chunking (of reference points) in delayed recall as was classically found with chess pieces could be investigated (elements belonging to the same chunk should be retrieved together temporally, with pauses between chunks). Knowledge elicitation interview protocols may be developed for this, on the basis of track maps (Racing drivers should be familiar with using these as memory recall aids, as they are used in technical debriefings with engineers). Verbal commentary of video replays may also be useful. Drivers should be asked to annotate a track map/comment on a video, and the order and timing of retrieved content in the protocol analyzed to determine chunks. Also, higher-order semantic associations could be coded (logical or causal dependencies of actions at different parts of the track).

One caveat—which applies to all domain expert knowledge elicitation and protocol analysis—is that this method may be limited by what information the experts are able to access *explicitly*. Reference points (and waypoints especially) may be quite *implicit*, and the expert may not be able to describe in detail, in words, all the reference points they are using (Here chess is different as the pieces establish as “base level” of discrete physical features one can always readily point to—and they are the same for everybody). Clever ways to set up structured interviews and protocol analysis procedures to shed a light on what the items in the chunks are would be desirable, and theoretically interesting.

#### Performance, Physiology and Brain Function

Given the limits of explicit knowledge and verbal reports, more direct ways to probe reference point memory and waypoint-directed action (and the predictive processing of unseen reference points) should be developed on the basis of behavioral, physiological, and neuroimaging measures.

Visual orientation to waypoints (timing of saccades, fixation locations) can be obtained through eye tracking. Because waypoints are operationally defined in terms of where control actions occur, this information can be gleaned directly from simulator telemetry data or from race car and GPS localization data in the field. In other sports, foot placement (e.g., long jump, cf. Lee et al., 2009), grips in rock climbing, or gaze landing points could be used as operational criteria for waypoint actions and locations; in the abstract sense, a waypoint specifies when and where the control surfaces of the body/vehicle “make contact” with the environment.

The distribution of waypoints in space and time, and how this relates to variation in performance within and between drivers should be investigated. An interesting question is the role of variability in waypoint locations, and how this variability relates to performance. Waypoint locations need not be exactly the same spot every time. They will not be fixed in the scene at the long time scales reference points are. The “perfect line” is *not* the same trajectory (navigation level) each time. The exact positioning of waypoint locations will depend on the action plan (top-down) *and* the current dynamic state (feedback, motor program, bottom-up) as well. Part of expert skill probably lies in how their location may be subtly adjusted lap-by-lap, on the basis of sensing the dynamic state of the vehicle/body.

The current hypothesis predicts that *reference point* localization accuracy should be monotonically related to performance (increasing), but the relationship with *waypoint* location variance and performance relationship might be nonmonotonic (concave): increasing skill reduces “human error” variability, but still higher levels of expertise may be reflected in non-metronomic, “flexible” variability.

With eye tracking one may also probe how, where and when reference points are read into memory. Whether and how waypoints may be designated by gaze (i.e., the “waypoint identification hypothesis” could be studied). The information that can be gleaned is limited, however, by the fact that multiple reference points and waypoints chunked simultaneously necessarily involves peripheral vision (Wolfe et al., 2017; cf. Vater et al., 2020) and covert attention. Phenomenologically, racing drivers describe this use of peripheral vision as maintaining the focus of attention “expanded” (Bentley, 1998, p.105). This “widescreen” visual awareness is one of the most distinctive features about how racing drivers describe their use of vision and merits further investigation.

One possible paradigm would be to allow familiarization of a track and then to manipulate the availability (or the visual features or locations) of reference points. For example, in a simulator, the 3D layout of a familiar track can be reconstructed, but the textures and patterns that serve as landmark features can be modified. Effects on performance can then be analyzed (performance engineering can be used to determine meaningful performance measures). If driving performance was based simply on road geometry sampled at the point of gaze, then changes in peripheral detail should not matter. If the driver were sensitive only to the available sensory visual input (retinal image), changes to landmark texture or other features should not matter. But if the driver is mentally associating the road preview to the entire scene layout in memory and in peripheral vision, then they should. Besides effects on performance, learning, memory recall, and physical measures of prediction error should be studied. The latter include pupil dilation, saccades to the “surprising” reference points, skin conductance responses, or even EEG measures such as error-related negativity.

Covert attention could also be studied by establishing suitable EEG indices. The use of physiological measures should probably begin with more simplified, but still representative, tasks (moving to high-fidelity simulators and field experiments once the task and signals are understood, and robust analysis pipelines established). One such task would be encoding an array of visual features or locations, and then dynamically shifting covert attention among them (“widescreen” visualization). EEG could be used to probe the neural basis of this guidance-level sense of space.

To the extent that the simplified tasks indeed probe the same cognitive processes as the domain of expertise, experts should show marked superiority to control subjects on those tasks (only). This kind of between-subjects comparison can be used as partial ecological validation of task representativeness. Physiological differences between subjects, especially if correlated with task performance, would shed further light on the neural basis of such differences (e.g., EEG spectral analyses: alpha desynchronization associated with covert attention, theta synchronization with encoding and shifting task set, etc.).

fMRI would be suitable as well. Here in particular the interpretation of the results could draw on the rapidly advancing literature on the neural basis of visual scene analysis, wayfinding, and cognitive maps. The use of ever more dynamic scenes (like driving scenes) allows scientists to probe the role of time and the difference between viewing static snapshot pictures of a scene and moving through a scene (time representation, active sampling which are essential features of real-world scene analysis).

Particularly illuminating could be the work on a parallel form of navigational skill in expert drivers: London cabbies (Spiers and Maguire, 2006, 2007; Griesbauer et al., 2021). While vehicle dynamics, performance measures, and environment scale/3D complexity are different in these two domains of expertise, the design of the human memory system is the same, and therefore many of the computational problems and strategies may be the same.

Using knowledge of a large-scale allocentric space that extends beyond the field of view implicates the hippocampus and the retrosplenial cortex, and landmark recognition and visual scene analysis relies on temporal and parietal place areas (Barry and Burgess, 2014; Epstein and Vass, 2014; Spiers and Barry, 2015; Nau et al., 2018; Peer et al., 2020). The need to translate between points of vantage needs the kinds of multiple coordinate transformations associated with the posterior parietal cortex (Crawford et al., 2011; Lappi, 2016). Sustained attention (executive control, monitoring, task maintenance, and task switching) also implicates the premotor and (dorsolateral) prefrontal cortex, i.e., attention and salience networks, especially the dorsal attention network (Corbetta et al., 2008; Menon and Uddin, 2010; Menon, 2010; Scolari et al., 2015).

In terms of novice-expert individual differences, the waypoint chunking model would predict that for novices and for most “merely experienced” everyday drivers, the driving task could be more a case of following a visually designated path—but for racing experts “the racing line” would instead be based on memory encoding/recall (reference points) and more complex sub-goaling (waypoints). This would imply the involvement of brain networks beyond the “driving network” identified in meta-analysis of driving-task fMRI research (Navarro et al., 2018, and largely coinciding with the list of brain areas above). Where in the brain might these extra networks be? Here the sub-goaling the desired path into waypoints as a *chunking* process would suggest the (lateral, including *ventrolateral*) prefrontal cortex (The potential confound from using verbal code e.g., to self-monitor should be addressed here, though).

Encoding a scene into cognitive maps, and the retrieval of large-scale spatial structure and route/goal information are probably associated with circuits in the parahippocampal/hippocampal systems and the retrosplenial/posterior parietal cortex (possibly orchestrated by the prefrontal cortex). The retroplenial complex in particular should be the key hub that mediates the “chunking” that translates between the stable, fixed, allocentric, and dynamic, egocentric representations of reference points (Epstein, 2008; Vann et al., 2009). Indeed, it has been shown to be activated when racing drivers (but not control subjects) view in-car footage, and its size correlates to real-world racing performance (Bernardi et al., 2014).

The level of track familiarity and driver skill should modulate brain circuit activations, according to their roles in encoding new chunks into long term memory vs. retrieval of long-term memory information. Whether or not “binding” spatial/action chunks relies on the same networks as the binding of *object* features into chunks—studied in most working memory and visual search experiments—deserves due consideration. Another type of binding process that may be more relevant (in case the mechanisms are different) is the “grouping” targets in multiple-object tracking tasks (Yantis, 1992; Cavanagh and Alvarez, 2005; Scholl, 2009; for brain imaging on such tasks see Nummenmaa et al., 2017).

### Generalizability to Expertise in Other Sports

Developing the ideas on the case of the motorsport athlete has allowed a fairly clear and empirically grounded task analysis. However, none of the core concepts, assumptions, or the principles derived depend on the specifics of motorsport, such as on using a vehicle for locomotion, using steering wheels and pedals to control the vehicle, the use of engines to produce motility, the use of tires to generate control forces on the ground, asphalted tracks that loop in on themselves, etc.

They should be applicable to any task (sport) with similar task demands. The core aspects of the task are:

(1) *Visual guidance and control* of one’s trajectory (path of the vehicle or one’s body) with respect to a stable environment.
(2) Performance advantage that comes from “fixing” in one’s mind a clear picture of the 3D scene layout…
(3) …at both the navigational level (allocentric, stable cognitive map) and the guidance level (egocentric, dynamic widescreen awareness of the visual field), requiring complex coordinate transformations.

These skills, according to the theory, are major performance determinants in high-speed sports. That is, sports where perceptual-cognitive judgment, rather than physical capabilities of the body, limit performance, with travel speed as the relevant metric. Downhill skiing, mountain biking, drone racing etc. are clear examples, and would provide domains to study the hypotheses (Indeed, because of the economy of representation one would predict the higher levels need not even “know” what the mode of locomotion is; only the control level “knows” about steering wheels, pedals etc. This makes those higher levels potentially more flexible and transferable across domains).

To what extent the chunking hypothesis in its present form applies to, say, whitewater kayaking or surfing, where the *scene layout itself is constantly fluctuating*, is less obvious. Presumably, this type of environment affords fewer reference points, and performance is more dependent on the control-guidance level interactions (Whether and how waypoints might still be “fixed” with gaze is an interesting question).

Sports *lacking the high-speed element* such as rock climbing appear to fulfill all three core aspects above, however. So, achieving speed may not be an essential property, as other performance metrics might be used. The concept of a waypoint is quite general, and can be operationalized, e.g., by the placement and type of grip in climbing; the concept of a reference point and chunk likewise transcend domains.

In many sports relative motion between the observer and relevant scene elements is not caused (only) by observer motion, but by the *movement of the objects themselves in the 3D scenes*. Examples would be skeet shooting, football, or ice hockey. How one “reads” the scene in these situations may require additional assumptions, as there are relevant scene elements that are not reference points fixed in a 3D frame—i.e., their motion is more “random” (or: the correlations in apparent motion are different from the optic flow generated by self-motion).

Also, many games introduce additional rules, such as designating players as opponents and teammates, so the correct way to “read” and interact with the situation is based on *an additional layer of scene semantics*, rather than the scene layout and the physics of the situation. Many games also bring in an *adversarial* component (Which of course is also present in racing against opponents—the present theory only considers the more restricted core skill of driving).

## Conclusion

We are perhaps nearing the goal of tackling the neurocognitive basis of elite sports performance, “the brain’s biggest challenge” (Walsh, 2014). Neuroscience is poised to move forward from simple, sedentary laboratory tasks to more dynamic domains of expert performance. Machine learning develops concepts and methods that are able to take on increasingly complex dynamic tasks, achieving even superhuman performance. The challenge is to bring all this together, towards an understanding of human cognitive processes (which have a basis in the hardware of the brain and may be different from AI).

Here the domain of expert driving offers many desirable features, both in terms of methodological opportunities and the chance to build on a solid basis of existing concepts and theory. The aim of this article was to show how apparently disparate and often unconnected lines of inquiry can be unified by the integrative concept of chunking. In cognitive science and the psychology of expert performance this concept has for decades been the standard way to understand the cognitive basis of expert performance in domains such as chess but it has not been fully deployed to understanding sports, real-world skills, and expert performance in physical locomotion. It opens a way to bring elite sports performance into contact with the theory of chunking (traditionally used to analyze sedentary tasks like chess).

Another contribution is to show how the concepts of predictive processing can be used to (re)interpret the concept of chunking for skilled dynamic task performance. This may open up new and interesting ways to understand the nature and roles of different types of “cognitive representations” in sports, and potentially even the differences between human and machine information processing in complex dynamic tasks.

For the cognitive psychologist working in the field of expert performance, the egocentric chunking hypothesis offers a novel view of chunking in dynamic tasks. For the computational modeler in cognitive science and engineering, it may pave the way for more psychologically and neurologically plausible computational models for simulating human performance. Of interest to the development of future AI may be how humans are able to outperform AI in locomotor tasks requiring flexibility in the face of complexity—despite severe limitations in processing speed and attentional capacity. Especially when embedded into a predictive processing framework. For the neuroscientist, the theory suggests road map for translating basic concepts and theories (wayfinding circuits, predictive brain) into more complex dynamic tasks, representative of expert performance “in the wild”. For the practitioner, it may offer a way to translate their domain understanding to terms and definitions commensurate with cognitive psychology, computational cognitive modeling, and modern neuroscience.

The field of expert performance is ripe for developing theories and models of the perceptual-cognitive expertise of high-speed athletes, such as racing drivers. It is hoped that this article will stimulate researchers and practitioners in different fields to see connections between disciplines and that neuroscientists, psychologists, engineers, and computer scientists working on different aspects of the problem of human performance will find the theory useful, and then go on to develop more accurate, multidisciplinary theories and methodologies…and then put them to the test in the laboratory, in simulators, and out there “in the wild.”

## Data Availability Statement

The original contributions presented in the study are included in the article, further inquiries can be directed to the corresponding author.

## Author Contributions

OL conceived and wrote the manuscript and made the pictures.

## Conflict of Interest

The author declares that the research was conducted in the absence of any commercial or financial relationships that could be construed as a potential conflict of interest.

## Publisher’s Note

All claims expressed in this article are solely those of the authors and do not necessarily represent those of their affiliated organizations, or those of the publisher, the editors and the reviewers. Any product that may be evaluated in this article, or claim that may be made by its manufacturer, is not guaranteed or endorsed by the publisher.

## Footnotes

^1^A scientific theory cannot be tested on the evidence that has been used to develop it, so the use of a broad basis of observations from wide a range of sports to “support” the statements before the ideas are fully fixed would make *all* those observations inadmissible as evidence.

^2^The final outcome, *the egocentric chunking* model, should not substantially depend on the choice of task analysis framework; it can be regarded more as a kind of heuristic scaffolding.

^3^From any of this it does not follow that the allocentric map would need to be a Euclidean, metrically accurate 3D replica of the environment, however. See Section “Chunking in the Predictive Brain”.

^4^Sometimes in the psychological literature simply “cutting a corner” —i.e., moving to the inside edge of the bend which is a ubiquitous steering strategy also in everyday driving (Spacek, 2005)—is called “taking a racing line”. This however does not correspond to the actual geometry of the racing line taken by expert racing drivers (for an illustrative analysis of the racing line see Lopez, 1997).

^5^Lappi (2014) defines waypoints as “*any point on the visible path, such as a marking on the pavement, or, if the driver plans his FP [future path], a point such as the turn point (where the driver will turn the wheel entering the bend) … they need not in principle present any distinctive visual feature*.” Lappi and Mole (2018) consider waypoints *objects or locations* that are both *visual targets* (for gaze), and *locomotor targets* (for steering), proposing that it is the “*visual target acquisition and tracking system*” (the saccade and pursuit eye movement systems) that translates environmental locations into a form of representation appropriate for steering control, thereby making the visually targeted location a locomotor waypoint: the “*waypoint identification hypothesis*”. Note that the waypoint concept is quite different from *travel points*, such as the tangent point (Land and Lee, 1994; cf. also Land and Tatler, 2001; van Leeuwen et al., 2017; Lima et al., 2020), or “near and far points” in most psychological steering models (Salvucci and Gray, 2004), which move with the observer and are never intercepted.

^6^On blink control in race drivers see Nishizono et al. (2021).

^7^Under a Bayesian interpretation, feedback (prediction error) is *data* is combined with *priors* and *likelihoods* to update the prior belief state into a *posterior* belief state. Bayesian inference process combines the old and new information in a way that takes into account their uncertainties. When the internal models are uncertain in the context and sensory information is precise, then the new sensory information (prediction error) dominates the posterior belief state, but when the prior information is strong and input is noisy or lacking, then prior information dominates. The posterior belief state then becomes the new prior information and the cycle continues.

^8^The higher levels on the motor side are action plans (decisions on the racing line, such as how to turn into the corner and whether to take a wide line, or to clip the apex).

^9^Communication skills are also needed at the expert level, to work with engineers, but they are a distinct skill set from the core car-control and driving skills often developed in childhood.

^10^100,000 chunks to reach master level, which, with some reasonable assumptions about the learning rate, also yields the 10,000 h/10 year “rule” for the acquisition of expertise (Ericsson et al., 1993; Ericsson, 2006) also consistent with biographical material (Bloom, 1985). All these figures should be interpreted in terms of suggestive *order of magnitude* only.
